# Alternate Access to Care: A Cross Sectional Survey of Low Acuity Emergency Department Patients

**DOI:** 10.7759/cureus.1385

**Published:** 2017-06-23

**Authors:** Jacqueline MacKay, Paul Atkinson, Erin Palmer, Jacqueline Fraser, Elise Vaillancourt, Michael Howlett, George Stoica, Maria Powell

**Affiliations:** 1 Family Medicine/Emergency Medicine, Dalhousie University; 2 Emergency Medicine, Saint John Regional Hospital; 3 Family Medicine, Saint John Regional Hospital / Dalhousie University; 4 Emergency Medicine, Saint John Regional Hospital / Dalhousie University; 5 Research Services, Horizon Health Network

**Keywords:** primary care, advanced access, patient acuity

## Abstract

Introduction

Patients with low-acuity (Canadian Triage and Acuity Scale level IV and V) complaints use the emergency department (ED) to access care. This has often been attributed to lack of a primary care provider. However, simply being registered with a primary care provider may not prevent low acuity ED presentation. There is some evidence that a lack of timely access to primary care may contribute to low acuity ED presentations. The Wait Time Alliance, a group of Canadian physicians and their respective professional associations, has recently set a benchmark of same day access to family doctors. It is unclear if this benchmark has been achieved in all jurisdictions.

Methods

We performed linked cross sectional surveys to quantify the number of people presenting to the ED for nonurgent problems who felt unable to access primary care. Primary care practices were also surveyed to assess access using the metric of time to third next available appointment.

Results

In the patient survey, 381 of 580 patients consented to participate. Of the 89 patients who met eligibility criteria, 100% completed the survey. 32 (35.9%) reported that the wait to see their primary care provider was “too long”. 45 (50.5%) patients did not contact their primary care provider’s office prior to ED presentation. 45 of 72 physician surveys were returned; a response rate of 62.5%. Most (77%) physicians estimated their wait time for a standard appointment to be greater than 48 hours. The mean calculated time to third next available appointment in the region was 6.6 (95% CI 4.6-8.7) days.

Conclusions

Approximately half of low acuity patients do not attempt to access their primary care provider prior to ED presentation. The benchmark of same day access to primary care has not been achieved in many practices in our region. Further education regarding primary care access would likely be beneficial to both patients and providers.

## Introduction

There is a reported problem with timely access to primary care in Canada. A 2014 Commonwealth Fund comparison of health care systems around the world ranked Canada eleventh out of eleven nations in most measures related to timeliness of care [1]. In 2011, only 51% of those surveyed stated they could access a doctor or a nurse on the same or next day the last time they required medical attention [1]. In 2013, 62% of respondents rated it difficult to get after-hours care without going to an emergency department (ED). In response, the College of Family Physicians Canada endorsed the Wait Time Alliance recommendation in 2015 to define same-day access as the Canadian standard for access to primary care [2].

Regular use of emergency departments (EDs) for low-acuity complaints has often been attributed to lack of primary care, particularly by patients with no regular primary care provider [3]. It was noted that 41% of Canadians surveyed in 2011 reported that they visited an ED for a condition they felt could have been treated by a primary care provider, had one been available [1]. A recent review of ED use in eastern Canada found that a large proportion of patients presenting to the ED actually had a documented primary care provider, including those who used the ED frequently (four or more visits in one year) [4]. Simply being registered with a primary care provider may not prevent low acuity ED presentation: there is evidence that better actual access to primary care is associated with decreased ED use [5].

‘Advanced access’ is a system of clinical practice redesign adopted in many provinces in Canada to reduce delays in primary care access by implementing same-day appointment scheduling [6]. Essentially, advanced access is a scheduling process redesign that allows patients to access primary care within 24 hours of the request to be seen [7]. Research has shown that populations with better access to continuing care from the same physician have fewer hospitalizations and better outcomes [6]. The College of Family Physicians Canada, therefore, recommended that primary care practitioners consider practice redesign tools such as advanced access, and that system supports should be in place to implement advanced access [6]. Some provinces have initiatives to train all primary care providers in advanced access, but there is no such initiative available to primary care providers in New Brunswick [6,8]. In provinces supporting clinical practice redesign, participating practices were able to reduce wait times to less than two days [8].

Rationale

Patients attending the ED for nonurgent complaints in the study area may not be able to access their primary care provider in a timely fashion. The purpose of this study is 1) to determine reasons for ED presentation in patients who have low-acuity complaints despite having a primary care provider; 2) to document patient perceptions of primary care access in this cohort; 3) to assess primary care access in our region and determine if it meets the Canadian benchmark of same-day access; and 4) to compare patient stated access to actual access metrics provided by primary care offices.

## Materials and methods

This study consisted of two cross-sectional surveys: one for patients attending the ED of a tertiary care centre with low acuity presenting problems (patient group) and one for primary care providers in the same urban area (physician group). This study protocol was approved by the Horizon Health Research Ethics Board (HHN REB#: 2015-2114).

### Participants

This study was carried out in the Saint John area in New Brunswick. Patients presenting to the ED of the tertiary care centre triaged as Canadian Triage and Acuity Scale (CTAS) levels IV and V, who reported a primary care provider, and did not have an acute injury such as a laceration or a fracture requiring ED resources were included in the patient group. 

All primary care practices registered with the regional Department of Family Medicine were asked to participate in the investigation of accessibility to primary care in the physician group.

### Materials

A printed survey was administered to appropriate consenting patients in the ED (see appendix). A printed survey was administered to all family physicians in the local area by email, mail, and by fax, if necessary (see appendix).

### Procedure

Patient Group

To determine patient perception of access to primary care, a survey was administered to patients in the ED waiting area. Data was collected during the hours that the Rapid Assessment Zone (a specific low-acuity area) of the ED was open in a sample of convenience. The survey was offered to patients presenting to the ED with a cover sheet that explained the reason for the survey, obtained consent, and screened for those who met the inclusion criteria with the aid of the triage nurse and registration staff, while maintaining patient confidentiality.

Physician Group

Prior to data collection, all affiliated primary care providers were assigned a number to facilitate blinded data collection and comparison.

In order to quantify access in the Saint John region, a brief questionnaire was administered to family medicine offices. This survey collected some data regarding practice design and the calculated metric of time to third next available appointment. In collaboration with the Department of Family Medicine, a brief explanation of the study and a copy of the survey with instructions on calculation of time to third next available appointment was emailed and mailed to all associated primary care practices. Two mail-outs occurred approximately 2 months apart; self-addressed, stamped envelopes were included to facilitate survey return. Unreturned surveys were followed up by phone and a survey was faxed to the physician’s office if a secretary could be reached. Physicians not practicing primarily outpatient family medicine were excluded from the sample group. Physicians with a mixed practice such as nursing home coverage or urgent care shifts were included in the survey. 

Physicians were asked to estimate the average wait time for a standard office appointment. Their office staff were also asked independently to calculate the actual time to third next available appointment. This was reported separately from the physician-estimated wait time in order to compare a physician's estimated wait time to their calculated wait time using a standardized measure. 

The metric for quantifying primary care accessibility endorsed by the advanced access system, the time in days to the third next available appointment, is felt to be a true reflection of standard office appointment wait times, as the first and second next available appointments may be due to cancellations or no-shows [7]. Calculation of the time to third next available appointment is simple and can be done manually by a medical office assistant. In order to calculate this metric, the number of days between a request for an appointment with a physician and the third next available appointment for a new patient physical, routine exam, or return visit exam is counted on the appointment scheduling system. Calendar days including weekends and days off are included, while saved appointments for urgent visits are not, as they are “blocked off” on the schedule [9]. 

### Data Analysis

Data collected from this study was analyzed using descriptive statistics to determine if there is any correlation between wait times and low-acuity ED presentations. The results of the survey performed in the ED and the information gathered from primary care offices were used to examine if there is a difference between patient perceptions of access and provider-reported access. Time to third next available appointment was averaged across the physician group to determine if access met the Canadian benchmark. Pearson’s correlation was used to see if there is a relationship between physicians’ estimated and calculated wait times. 

## Results

Data was collected in the ED and in community primary care practices between June and December of 2015. 

Patient Group

A total of 580 patients presenting to the ED were approached and asked to participate in the survey. Of those patients, 381 consented and completed the survey (65.7%). 143 patients were Canadian Triage and Acuity Scale (CTAS) level IV or V. 154 patients were assessed by the triage nurse as being safe to be seen in a primary care office. (Of those 154, 50 patients were triage level CTAS III or higher.) 89 patients had a primary care provider (PCP), therefore meeting all three inclusion criteria (15.3%; Table 1). All eligible participants completed the survey (100%).

**Table 1:** Patient Characteristics

	Attempt to call PCP (%)	Consider after hours clinic (%)	Age (mean & range)	Female (n, %)	Male (n,%)	Gender Unknown (n, %)	EducationHigh School or below (n,%)	Education College or Trade School (n, %)	Education University Degree (n,%)	Education Unknown	Employment Full time or part time (n, %)
Eligible Patients (n = 89; 5 incomplete)	46% (n=39)	28%(n=25)	38.5 (0-90)	41 (46%)	42 (47%)	6 (7%)	36 (40%)	33 (37%)	16 (18%)	4 (5%)	45 (50%)
Ineligible Patients (n=292; 47 incomplete)	22%(n=66)	27%(n=78)	44 (0-103)	131 (53.5%)	108 (44%)	5 (2.5%)	107 (44%)	90 (37%)	33 (13%)	13 (5%)	103 (35%)

**Figure 1:**
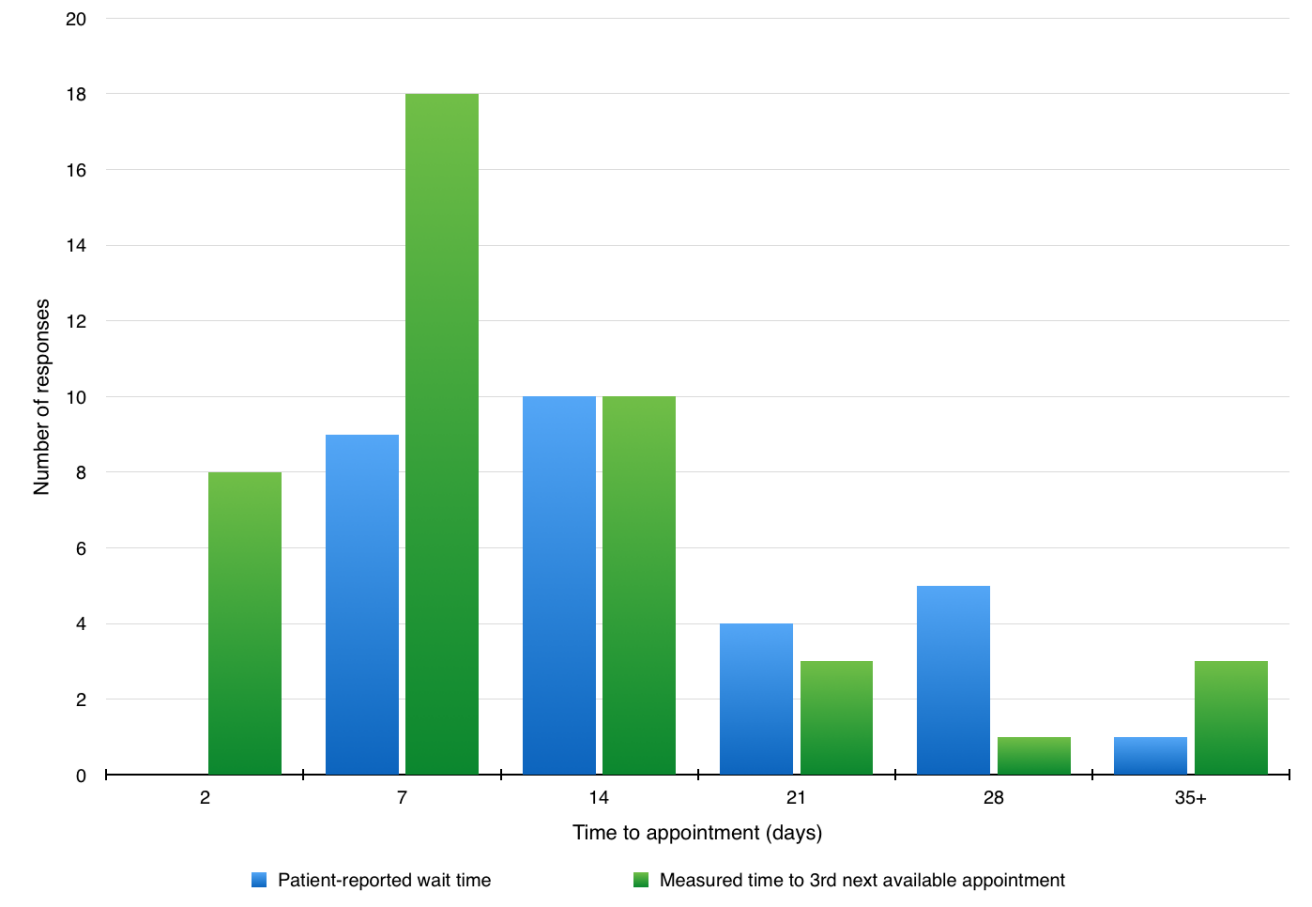
Patient Perception vs. Measured Access

Of the eligible survey participants, 45 (approximately 54%) did not attempt to call their primary care provider’s office prior to presenting to the ED for their low acuity complaint. In those that did call their primary care provider’s office, in 32 cases the phone was answered and one person was offered a same day appointment. In 20 instances, the patient reported a wait time of seven days or more (Table 2). Patient perception of access was compared to measured access in a graphical format (Figure 1).

**Table 2:** Patient-reported Wait Times to See PCP

Consented Patients (n = 381)	24-48 hours	3-7 days	7-14 days	14-21 days	21-28 days	More than 1 month
Eligible Patients (n=89; 29 responses)	0 (0%)	9 (31%)	10 (34%)	4 (14%)	5 (17%)	1 (3%)
Ineligible patients (n=292, 38 responses)	8 (21%)	9 (24%)	11 (29%)	5 (13%)	2 (5%)	3 (8%)

**Figure 2:**
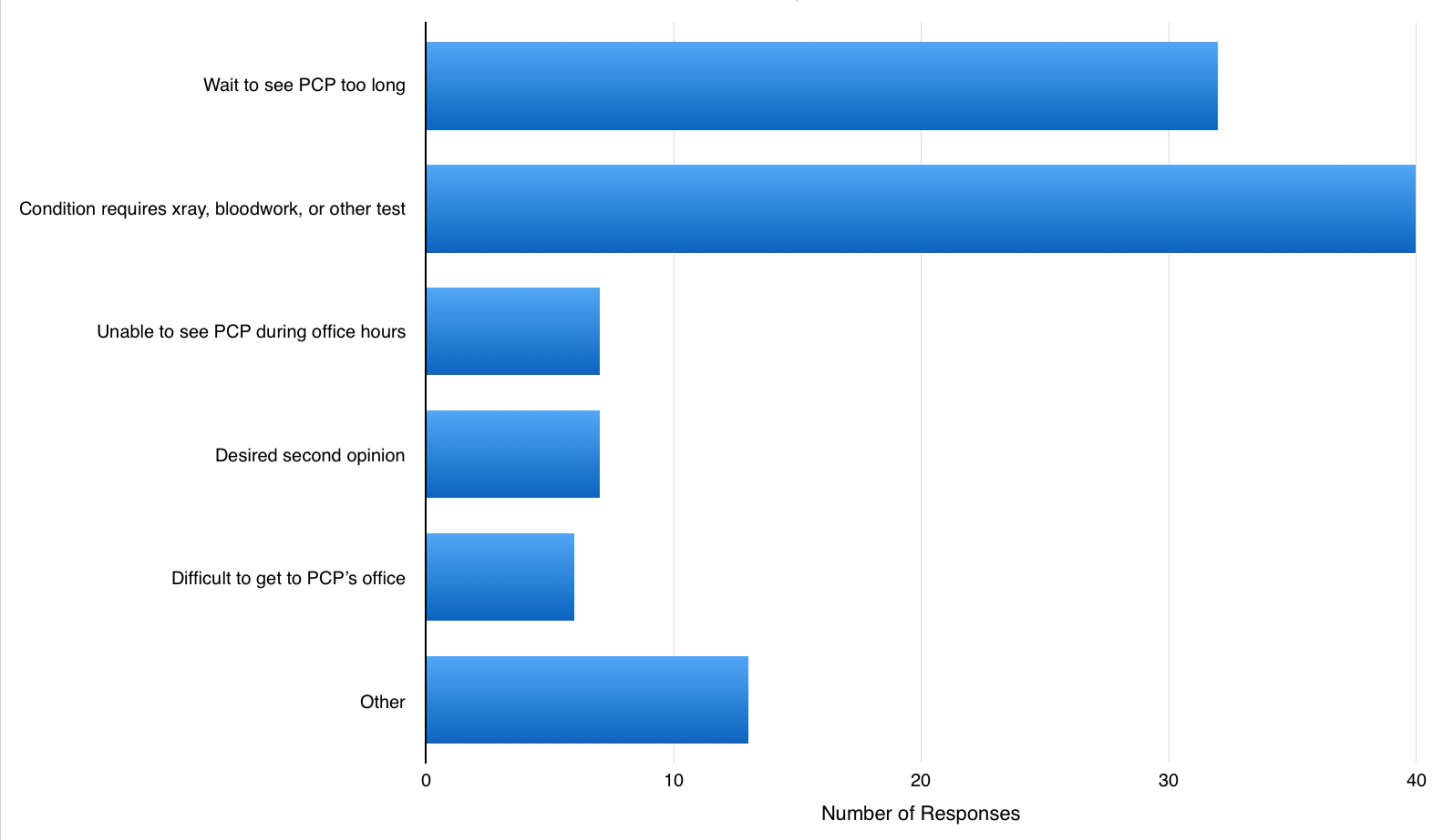
Reasons for ED Visit

Patients were able to report more than one reason for their ED visit. Of the eligible patients, 32 (36%) reported that reason for their ED visit was that the wait time to see their primary care provider was too long. 40 patients (45%) reported that they felt their condition required blood work, imaging, or other important tests (Figure 2). Group sizes were too small to comment on any patient characteristics that may contribute to low acuity ED presentation in patients registered with a primary care practitioner. 

Physician Group

A total of 45 physician surveys were returned from 72 physicians, a response rate of 62.5%. The average practice size was approximately 2000 patients, with a range of 500-5000 patients. The length of time in practice ranged from three months to 46 years, with an average of 15.25 years in practice and a median of 11 years. 

Physicians were asked to estimate their average wait times for a standard appointment. Many reported ranges such as 1-2 days or 1-2 weeks. One significant outlier (estimated wait time 42 days, time to 3rd next available appointment 50 days) was removed from the subsequent data analysis. The midpoint for the estimations was calculated. The mean physicians’ estimated wait time for an appointment was 8 (6.3-10.5) days. The mean time to 3rd next available appointment in the region was 6.6 (4.6-8.7) days. A patient can be seen within 24 hours of the request to be seen in four (9%) practices and with 48 hours of the request to be seen in ten (23%) practices. 

**Figure 3:**
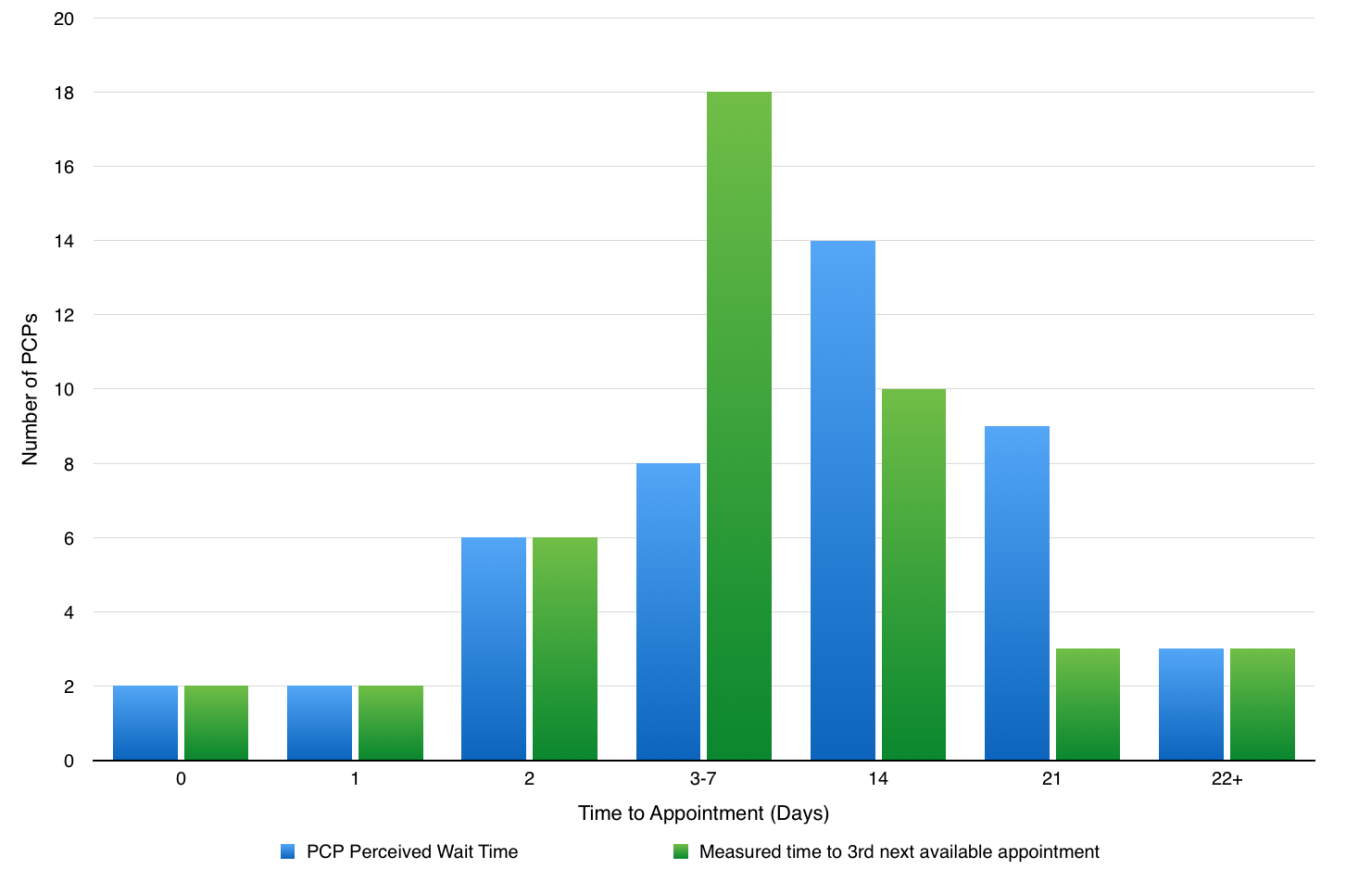
Wait Times: Practitioner Estimated vs. Measured Time

The majority (77%) of physicians estimate their wait time for a standard appointment to be greater than 48 hours. Pearson’s correlation was used to assess the relationship between physician’s estimated wait times and their calculated time to third next available appointment. The correlation showed a moderately positive relationship, indicating that physicians are accurate in estimating their wait times, compared to their individual measured access metric (Figure 3).

## Discussion

Low acuity emergency department patients constitute a small volume of the total use of hospital resources, and costs associated with the care of these patients are generally minimal. Though low acuity patients have been blamed for the ongoing epidemic of ED overcrowding, this group has little effect on ED length of stay or time to first physician contact for other, more urgent patients [10]. There has been no research to date on the relationship between the volume of low acuity patients and quality of care delivered in EDs. 

There is good evidence to support that patients who receive the majority of their care from a primary care physician have better health outcomes, particularly the elderly and patients with chronic diseases [11,12].Better continuity of care is also associated with decreased hospitalizations and therefore decreased overall system costs [13]. Many patients registered with a primary care provider would prefer to see their own primary care provider for both urgent and non-urgent concerns, if possible [14]. Thus, this group of patients registered to a primary care provider who presents to the ED with non-urgent complaints represents an important cohort in whom medical care could be optimized if they were seen by their primary care provider rather that in an ED.

In the patient cohort, more than half of non-urgent patients presenting to the ED did not attempt to contact their primary care provider prior to ED attendance. Despite this, many patients responded to the question regarding wait times for an appointment; this may be based on prior attempts to access primary care. All patients (with the exception of one offered a same-day appointment) reported a wait time in excess of 48 hours to see their primary care provider; 36% of patients reported that their reason for attending the ED was that the wait to see their primary care provider was too long. Given that some providers do report that they are able to see patients within 48 hours, it may be worthwhile educating patients about other options to access care prior to ED presentation. 

Many non-urgent patients also reported that they felt their condition needed hospital resources administered through the emergency department, such as blood work or imaging studies. Patients who were likely to require imaging, sutures, blood work or similar were excluded from the study sample at triage. It seems as though patients felt they required hospital resources despite a trained health care provider deeming them as ‘safe for GP office’; this is consistent with many other studies reporting that misperception of need is correlated with non-urgent ED use [15]. Same-day access to primary care would provide the same rapid physician assessment as ED presentation with appropriate management of their presenting problem in an environment with much greater continuity of care.

The mean time to third next available appointment in the Saint John area was found to be 6.6 days. There was considerable variability in the reported time to third next available appointment: the range was 0-22 days. Ten practices reported a time to third next available appointment for 2 days or less; this represents 23% of primary care practices whose patients can access their physician within 48 hours of the request to be seen. Physician estimates of wait times correlated with measured access, demonstrating that physicians in the study area are aware of their availability (or lack thereof). Though some practices are more accessible, access in the study area generally does not meet the Canadian benchmark of same-day access to primary care.

The measurement of the time to third next available appointment does not include appointments reserved for urgent patients, in order to provide a more sensitive reflection of true appointment availability [9]. Time set aside each day for urgent patients (typically those who call in same-day) is known as the ‘carve-out’ model of appointment allocation. This does provide better access for the patients who are able to book one of these appointments; however, it may rely on sometimes inaccurate triage for appointment distribution. Patients who are unable to book one of the reserved spots might attend the ED for prompt access to a physician, rather than making repeated attempts to book a reserved urgent appointment or wait for a routine appointment with their own primary care provider [7]. In our study, several physicians reported using a ‘carve-out’ model, which may have resulted in underestimation of access when time to third next available appointment was calculated for those practices.

Several Canadian provinces have been leaders in primary care reform. Many of these leading provinces have implemented and funded organizations with a mandate to support primary health care improvement and innovation. These organizations in turn support training programs for providers, development of inter-professional healthcare teams, and quality improvement strategies for system redesign at the practice level [16]. These voluntary and diverse initiatives have resulted from a collaboration between policymakers and providers and target healthcare infrastructure organization, healthcare quality and safety, and provider payment. Financial incentives and different payment schemes have had a positive effect in improving many measures of quality of care, including access to care and physician productivity. These differing payment schemes allow providers to implement practice changes to align with health system goals, supporting the development of infrastructure such as electronic health records implementation and encouraging the provision of priority services [16].

## Conclusions

This study surveyed low-acuity emergency department patients and primary care providers to assess access to primary care and reasons for low acuity unscheduled hospital attendance. Findings showed that many patients did not call their primary care provider prior to ED attendance; many patients reported that the wait time to access primary care was more than seven days. Patient perceptions of access to primary care and practice reported access appeared to be congruent, though this is not statistically significant. 

The Canadian benchmark of same-day access to primary care was met by only four out of 44 responding practices in the Saint John area. The mean time to third next available appointment was 6.6 days. This study demonstrates the compelling need for patient and provider education about access to primary care to improve health outcomes. 

Strengths and Limitations

This study was the first in New Brunswick to explore the accessibility to primary care using a standardized and objective measure of appointment availability. The data generated from the surveys can be used as the basis for further study of issues surrounding access to primary care, particularly as provincial governments initiate primary care reform. 

The access reported by patients and the access metrics reported by physicians were not matched in time nor were the patients matched to specific primary care offices. It is possible that the primary care offices whose patients attend the ED for non-urgent care also did not complete the physician survey; it is unclear what effect that would have on the mean time to access primary care in the study area, patient perceptions of access, and the relationship between the two. Individual primary care practices should be encouraged to be aware of their own access metrics and introduced to methods of primary care reform to improve access, if that is what is desired by the practitioner. 

The surveys performed represent one moment in time, rather than a continuous estimate of time to access primary care. It is acknowledged that physician availability and patient access is variable and dependent on many different factors.

## Appendices

### Appendix 1: Patient Survey

Dear Patient;

The Emergency Department is conducting a study that will help us understand wait times in the emergency department and wait times to get an appointment with family doctors. Family doctors in the Saint John area and the Saint John Regional Hospital feel this is an important issue to study as it may inspire changes that could improve access to medical care. We would greatly appreciate your help in understanding why people visit the emergency room by completing a brief survey while you wait.

You are eligible to participate in this survey if you have a family physician. All information collected will be confidential and will not be linked to your hospital chart. Your participation in this survey will not affect your care in any way.

Please give this package to both the triage nurse and the registration desk. Afterward, show the research package to the person who gave it to you and further instructions will be given for completing the survey. When you are finished, please place the survey in one of the collection boxes around the emergency department.

Oral Consent: Is patient (or caregiver) agreeable to participate? YES or NO

Triage;

1. Please circle the patient triage level:  Level 3 or higher

                                                                        Level 4

                                                                        Level 5

2. After assessing the patient, could this visit have been seen in a family doctor’s office? If the patient requires sutures, lab work, or a procedure etc. please state no

            Please circle: YES     NO

Triage: please give packet back to patient

Registration:

3. Does this person have a primary care provider? Please circle    YES    NO

Registration: please give packet back to patient

Research Staff:

Check the following:

                                                                                YES                                                      NO

Triage level 4 or 5

Safe for GP office

Does patient have PCP?

If all 3 replies are yes, please proceed with survey

Patient Survey

Some questions about your visit today:

1. Did you try to contact your primary care practitioner’s office (family doctor or nurse practitioner) before coming to the emergency room today? (circle one):      Yes               No

            If yes, did somebody answer the phone at the office? (circle one):       Yes     No

            If yes, were you offered an appointment today? (circle one):     Yes      No

            If no, how long did you have to wait to get an appointment? (circle one):

             24-48 hours                          3-7 days                    7-14 days

            14-21 days                            21-28 days                more than one month

2. Why did you decide to come to the emergency room today?

            __The wait to see my doctor or nurse practitioner was too long

            __I think my condition requires an x-ray or blood work, or other urgent tests

            __I am unable to go to the doctor or nurse practitioner during their office hours

            __I desired a second opinion

            __It is difficult for me to get to the doctor or nurse practitioner’s office

            __Other; please describe:

3. Did you consider going to urgent care or an after-hours clinic? (circle one): Yes             No

Some general questions:

What is your age? ____ years and your gender (circle one): M             F

Who is your primary care practitioner? (Family doctor or nurse practitioner):

4. What is the highest level of education you have completed?

__Grade 1-9                                     __Some college/certificate, did not graduate

__Grade 10-12                                 __College, CEGEP, or similar

__High school diploma                    __Some University did not graduate

__Apprenticeship/trade school       __University

5. What is your current employment status?

            __Unemployed, looking for work                __Full time employee

            __Unemployed, not looking for work         __Part time employee

            __Self-employed                                         __Permanently unable to work

            __Employed in the home                            __I do not wish to answer

            __Retired                                                       __Other; please describe:

Please return the survey to collection box when completed.

Thank you for your help with this study!

### Appendix 2: Physician Survey

How many years have you been in practice?

How many patients are registered in your practice?

What would you estimate to be the average age of your practice?

What is the average wait time for a routine office visit in your practice?

Does your office have ‘same-day’ appointments for patients with urgent complaints?

If yes, how many ‘same day’ appointment spots are blocked off every day?

Calculation of time to third next available appointment:

Access your appointment scheduler, paper or computer calendar. Find the third next available regular appointment. This does not include “blocked off” same-day appointments or other reserved appointments. Calculate the number of days until that next appointment, including weekend and holiday days. When is the third next available appointment? What is the number of days until the third next available appointment?

Do you use a standardized triage system to distribute appointments to your patients?

            If yes, what triage system do you use?

What is your model of practice?

            Salary

            Fee for service

            Other; please describe:

What is your model that you use for access to patient appointments?

            “Carve-out” model

            Advanced access model

            Other; please describe

            Don’t know

Does your office use:

            Paper records                       

            Electronic Medical Record               

            Combination of EMR and paper records

            Other, please describe:
